# Discussion on Pessaries

**Published:** 1891-09

**Authors:** 


DISCUSSION.

DR. HADRA remarked that adhesions behind the uterus, or the existence of an active inflammation contraindicated the use of pessaries. If the uterus on being replaced slipped back at once, it meant that the adhesions were not very dense. He mentioned several methods of replacing a uterus bound down by adhesions. First, the Swedish method of massage ; second, manipulation of the uterus under an anæsthetic, with a hand inserted into the vagina, and third, the performance of laparotomy and separation of the adhesions under the guidance of vision. In —85 he recommended making strong traction on the cervix with a tenaculum, and with the other hand stretching or breaking up the adhesions on the posterior wall of the uterus. This was published in the journals, but attracted no attention. Lately it has been revived in Germany. The Doctor made mention of the operation of stitching the uterus to the anterior abdominal wall, and Alexander's operation to shorten the round ligaments in order in the bad cases to retain the uterus in position. He expressed his belief in the value of pessaries, but said that to use them properly it was necessary to thoroughly understand their action. They were adjuncts to the treatment rather than the treatment itself.

DR. DENTON said that every case was a law unto itself. On account of the differences in the dimensions of vaginas and uteri, to adapt pessaries necessitated the possession of the mechanical skill to measure the size of those organs, and that was something only a few doctors had. The etiology of the displacement, he insisted, should be sought for in all cases. He thought good resulted from pessaries properly used; and after stating his lack of success with them, attributed it to lack of the necessary mechanical skill. Was fond of the Hodge pessary and thought the McIntosh was useful in some cases. Was doubtful whether the intra-uterine stem should ever be used.

DR. POYNER objected to pessaries in general, on the ground that continuous pressure on any part would finally, by cutting off the blood supply, lead to atrophy of that part. He was accustomed to use only the stem pessary that received support from the hips.

DR. HARDY expressed faith in their utility. He liked the soft rubber inflated pessary. A pessary so large or badly fitted as to cut off the blood supply, of course should not be used. All his patients were directed to remove the pessary if it began to give pain.

DR. McCALEB stated that a pessary was not fitted in so tightly as to cut off the blood supply, but had some motion as the body moved. When a woman is reclining a pessary is idle and presses on a different spot to that when she is erect, and thus the parts have time to recuperate. He thought that he had obtained good results in all his cases, although he had been obliged to change the form of pessary very often. He had used the closed and open lever and air pessaries, and also the one made of copper wire coated with soft rubber, which admits of being bent to any shape desired.

DR. CUMMINGS thought that if pessaries were so valuable, such serious operations as Alexander's and the stitching of the uterus to the anterior abdominal wall would not be needed. Removal of the inflammation or congestion, breaking up of the adhesions, together with proper rest, would in his opinion cure most of the cases. In general he did not approve of their use.

DR. WOOTEN stated that it was intended to limit the discussion to the use of pessaries in posterior displacements. For a person to say that pessaries had no support, was to declare his ignorance of the whole subject. A pessary to correct a retro-displacement must have its length adjusted to the cul-de-sac, its curvature to the degree of displacement, and must have proper support against the pubic arch.

DR. CHRISTIAN believed that he had in general found them of value. In his experience he had received no complaints that they interfered with sexual intercourse.

DR. DAVIS while admitting their utillty in certain cases, said that he had failed to find use for them often. He agreed in the condemnation of intra-uterine stems. In certain cases where there is relaxation of the vagina and procidentia, he believed that external supports are necessary.

